# Supplementary material for: Structural polymorphism of ex-vivo ALECT2 amyloid fibrils revealed by cryo-EM
Source: Nat Commun. 2026 Apr 11;17:5108. doi: 10.1038/s41467-026-71223-3 (PMC13247027; doi:10.1038/s41467-026-71223-3)
Supplement: Supplementary file 1 — Supplementary Information [file 41467_2026_71223_MOESM1_ESM.pdf]

Structural polymorphism of *ex-vivo* ALECT2 amyloid fibrils revealed by cryo-EM

Shumaila Afrin[1 $], Binh An Nguyen[1 $], Virender Singh[2], Preeti Singh[1], Parker Bassett[1], Maja Pekala[1], Bret Evers[3,4], Christian Lopez[5], Yasmin Ahmed[1], Li Li[6], Raja Reddy Kallem[6], Andrew Lemoff[7], Christos Argyropoulos[8], Barbara Kluve-Beckerman[9], Lorena Saelices[1 *]

[$]These authors contributed equally.

Affiliations:
[1]Center for Alzheimer's and Neurodegenerative Diseases, Department of Biophysics, Peter O'Donnell Jr Brain Institute, University of Texas Southwestern Medical Center (UTSW), Dallas, TX, USA.
[2]SciKonnect and BioPatriKa, Ambala, India.
[3]Department of Pathology, University of Texas Southwestern Medical Center (UTSW), Dallas, TX, USA.
[4]Department of Ophthalmology, University of Texas Southwestern Medical Center (UTSW), Dallas, TX, USA.
[5]Department of Internal Medicine, University of Texas Southwestern Medical Center (UTSW), Dallas, TX, USA.
[6]Jerry H. Hodge School of Pharmacy, Texas Tech University Health Sciences Center, Dallas, TX, USA.
[7]Department of Biochemistry, University of Texas Southwestern Medical Center, Dallas, TX, USA.
[8]The University of New Mexico School of Medicine, Albuquerque, New Mexico, USA.
[9]Department of Pathology and Laboratory Medicine, Indiana University School of Medicine, Indianapolis, IN, USA.
** Correspondence to: Lorena Saelices Gómez, lorena.saelicesgomez@utsouthwestern.edu*

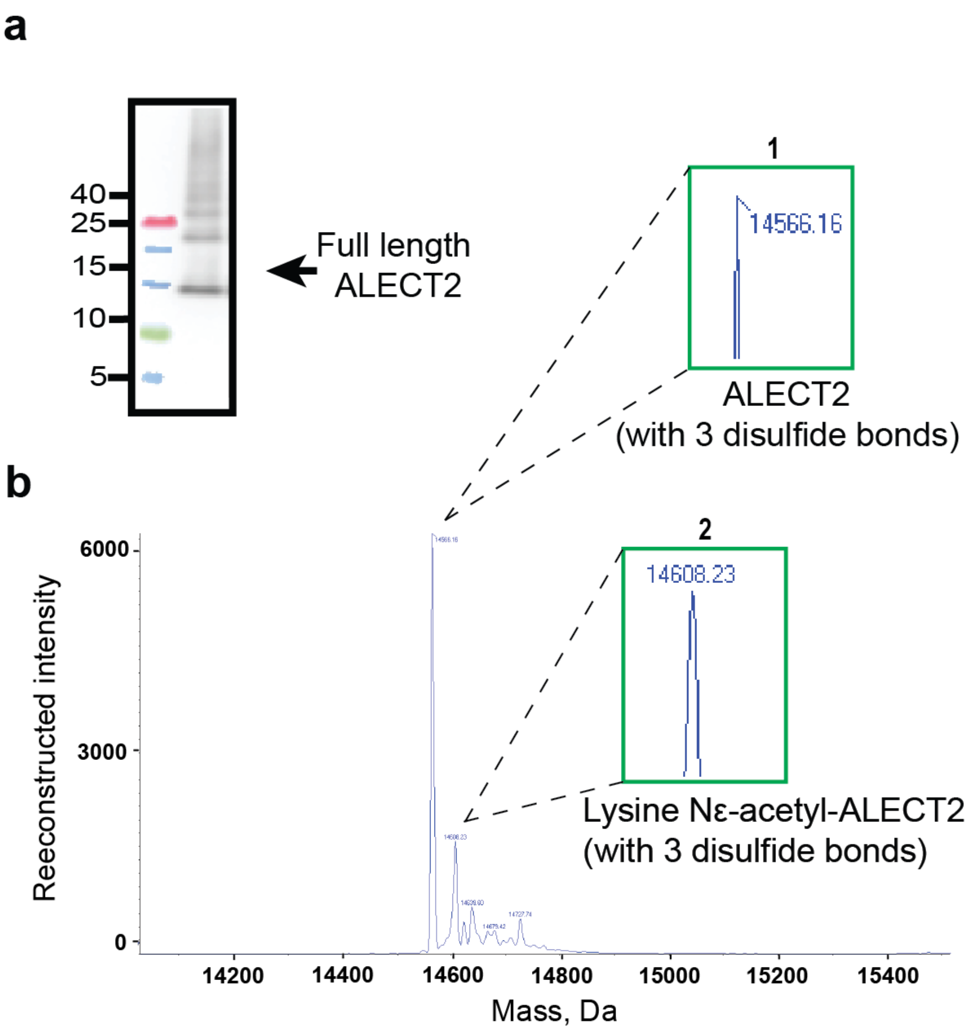

Supplementary Fig. 1 Characterization of ALECT2 fibrils extracted from the kidney. a Western blot of the water-soluble fibril elutions from the kidney using an antibody raised against full-length LECT2 protein. Arrow indicates band corresponding to full-length LECT2. Results for **a** representative of n=1 technical replicate. **b.** Intact Mass Analysis of ALECT2 fibrils extracted from the kidney**.** Deconvoluted mass spectrum of ALECT2 fibrils, confirming the presence of full-length ALECT2 with three disulfide bonds (peak 1). We detected additional post-translational modification (acetylation, peak 2) based on mass shifts in the spectrum. Source data provided as part of source data file.

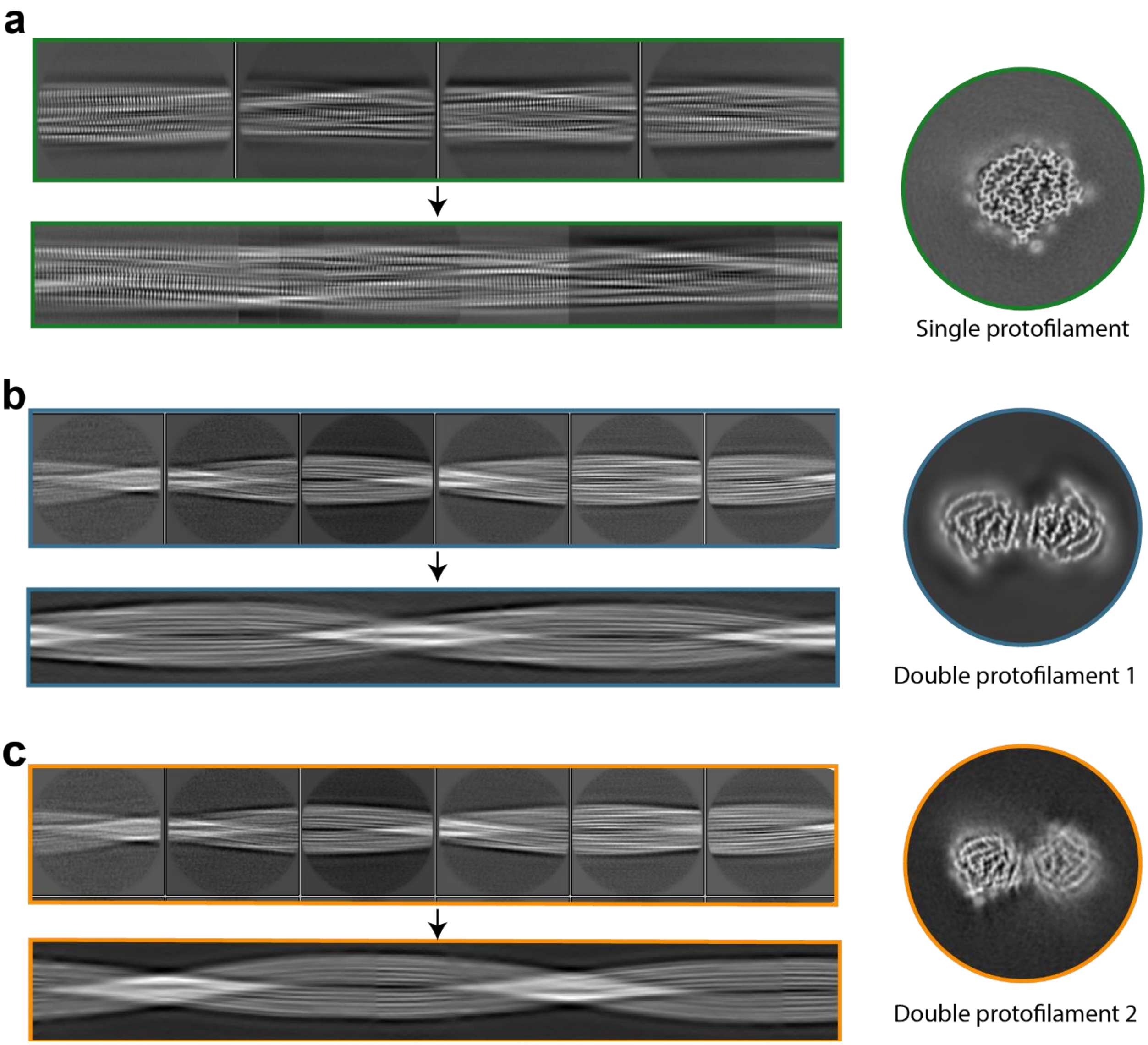

Supplementary Fig. 2. Representative 2D class averages and 3D reconstructions of ALECT2 single and double protofilament fibrils. **a.** single protofilament fibrils and **b.** double protofilament 1 fibrils and **c.** double protofilament 2 fibrils, extracted with a particle box size of 352 pixels. Below the individual 2D class panels, we present a stitched view of all 2D classes for the single protofilament fibril and the initial models of the two double protofilament morphologies, generated in RELION.

.

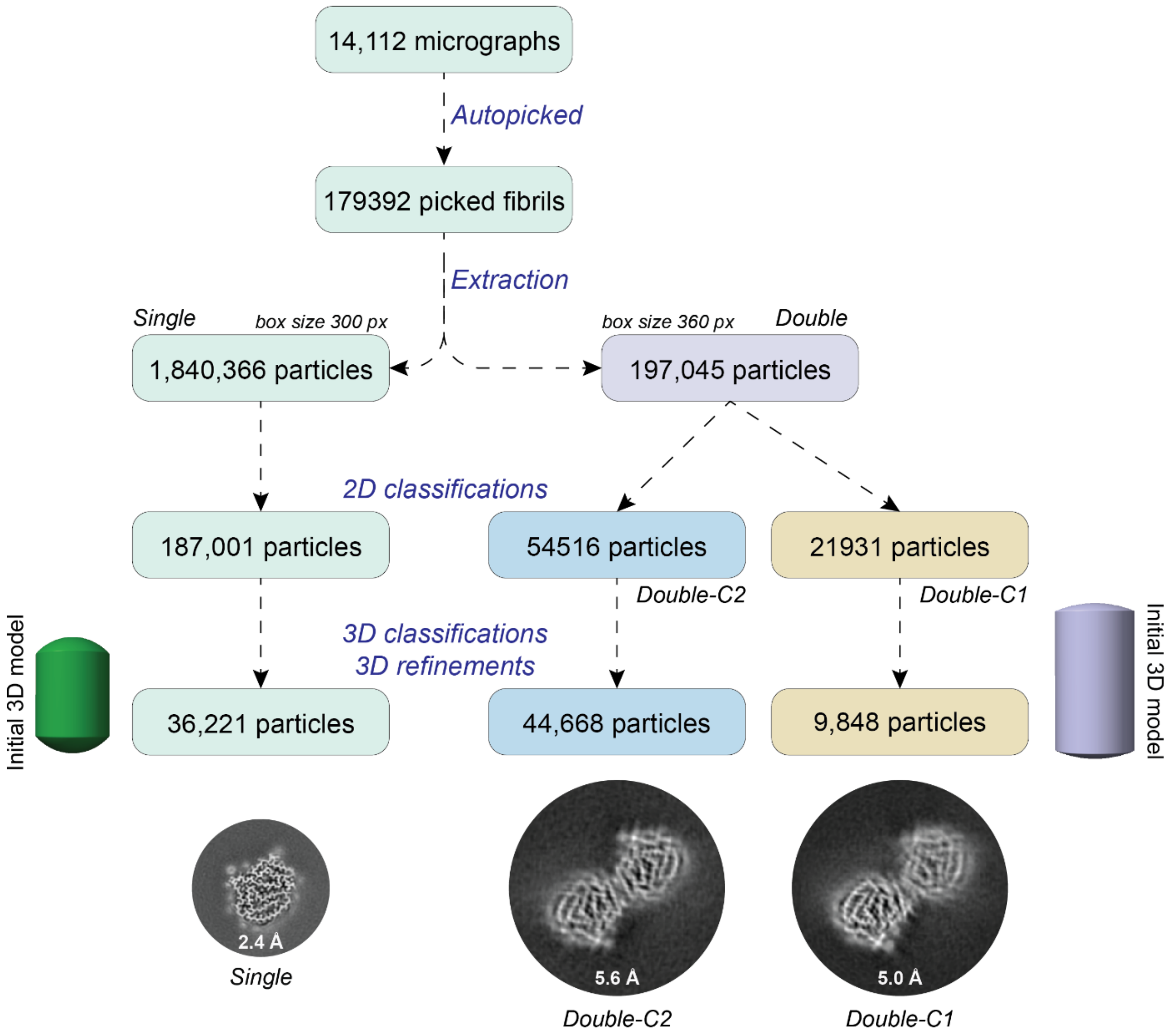

Supplementary Fig. 3. Cryo-EM data processing workflow for ALECT2 fibrils. A total of 14,112 micrographs were collected, yielding 179,392 autopicked fibrils. Particle extraction with box sizes of 300 px (single) and 360 px (double) produced 1,840,366 and 197,045 particles, respectively. After 2D classification, selected particles were subjected to iterative 3D classification and refinement using separate initial models, resolving one single-protofilament structure and two double-protofilament classes (Double-C1 and Double-C2).

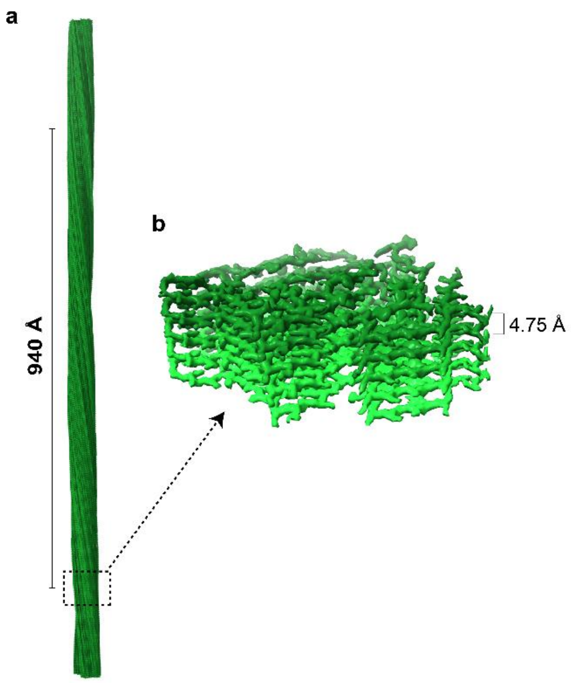

Supplementary Fig. 4. **Structural features of the ALECT2 single protofilament highlighting its helical parameters. a.** Side view of the reconstructed ALECT2 single protofilament model showing the crossover distance. **b.** The closeup side view of the map depicting the helical rise and layers.

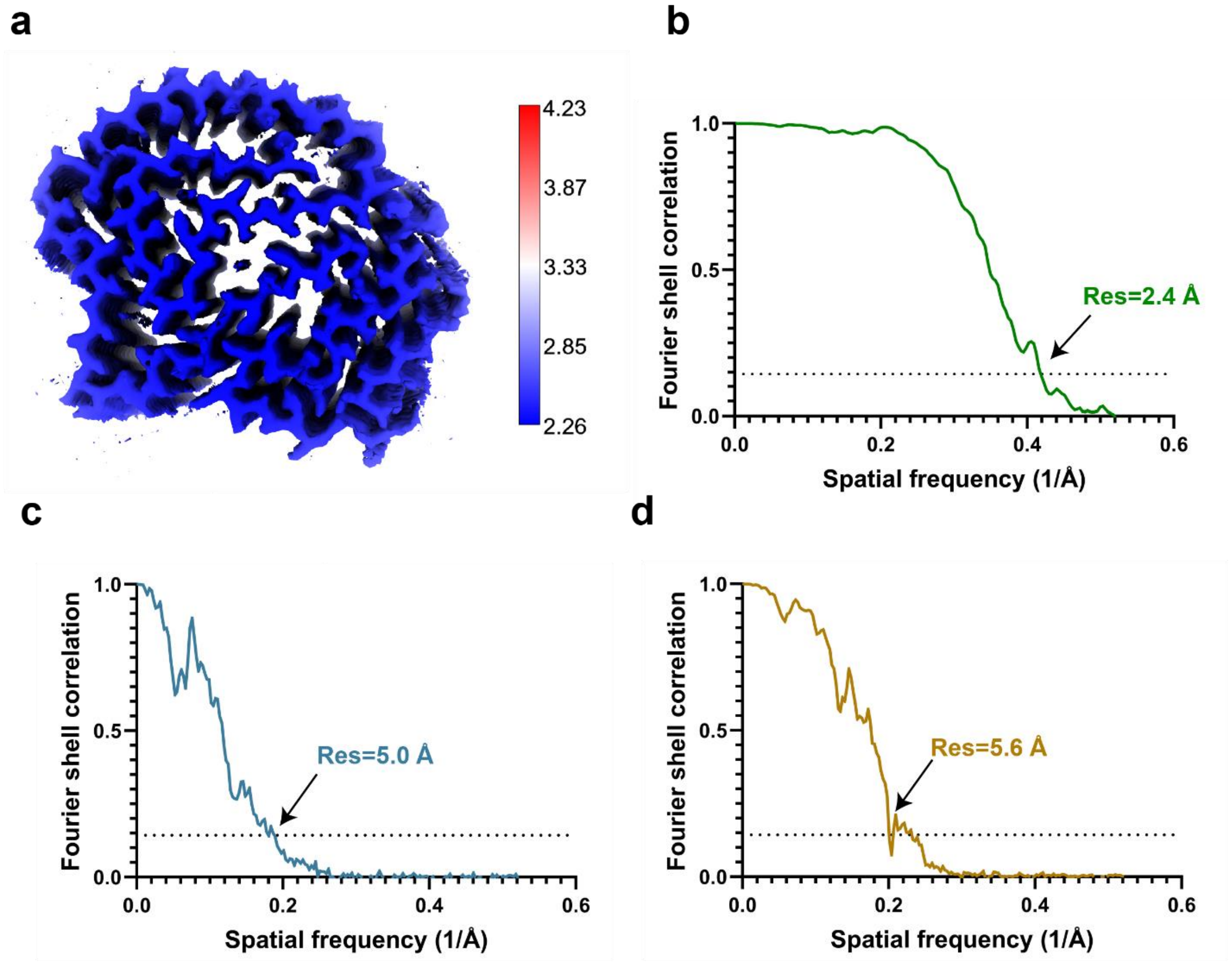

Supplementary Fig. 5. Resolution Assessment of the Cryo-EM Structure of ALECT2 fibrils from the kidney. a. Local resolution map of the ALECT2 fibril structure, highlighting variations in resolution across different regions of the density map. Fourier shell correlation (FSC) curve between two independently refined half-maps, illustrating the overall resolution of the reconstruction for ALECT **b.** single protofilament morphology, **c.** Double protofilament 1 morphology and **d.** Double protofilament 2 morphology.

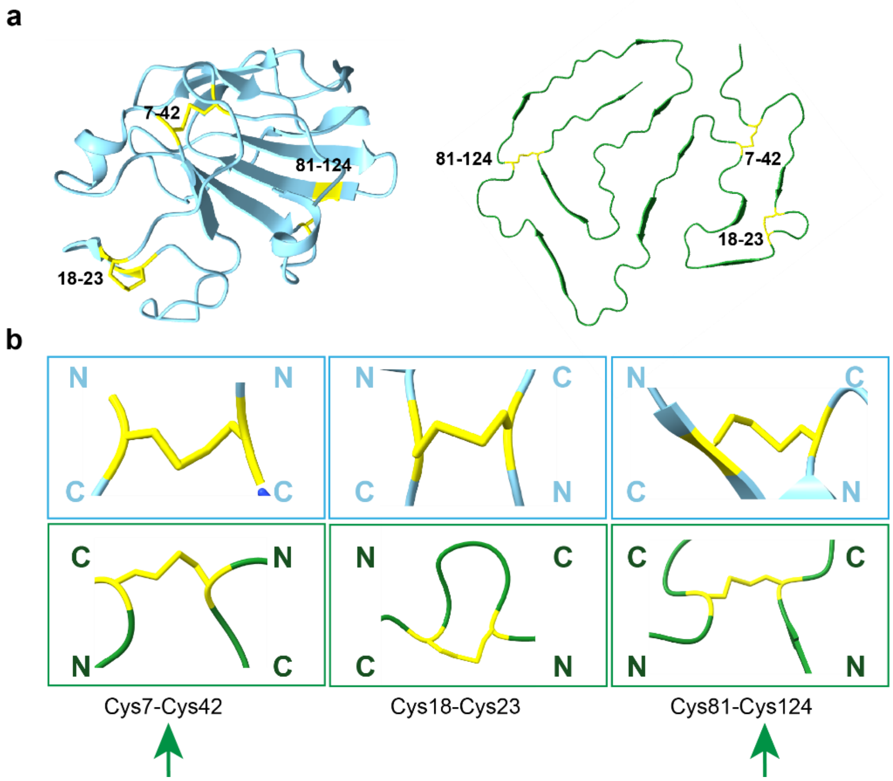

Supplementary Fig. 6. Structural reorientation of disulfide bonds in ALECT2 fibrils. a. Secondary structure visualization of native LECT2 monomer from the crystal structure (PDB ID: 5B0H, left) and ALECT2 fibrils from the kidney (right). Disulfide bonds are highlighted in yellow. **b.** Green arrows mark the disulfide bonds that change orientation. Cys7–Cys42 bond, which adopts a parallel N-to-C orientation in the native structure (light blue), shifts to an anti-parallel orientation in the fibril structure. In contrast, the Cys18–Cys23 bond maintains the same orientation in both native and fibrillar states. The Cys81–Cys124 bond transitions from an anti-parallel orientation in the native structure to a parallel orientation in the fibril.

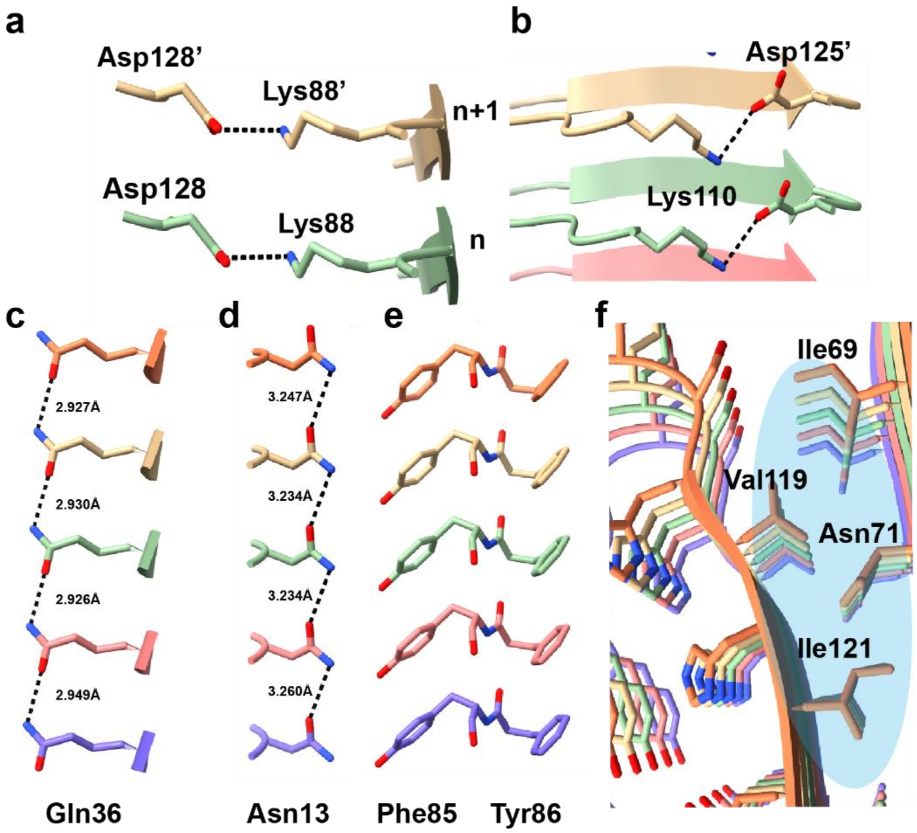

Supplementary Fig. 7. Additional interactions stabilizing the ALECT2 fibril structure.

a and b. Intra-layer salt bridge between Asp128 and Lys88 **(a),** and between Asp125 and Lys110 **(b)**. **c and d.** Network of hydrogen-binding polar ladders formed by Asn **(c)** and Gln **(d)** residues across all layers **e**. π-π interactions by aromatic residues across all layers represented here by Tyr 86 and Phe 85. **f**. Illustration of steric zippers stabilizing the fibril fold.

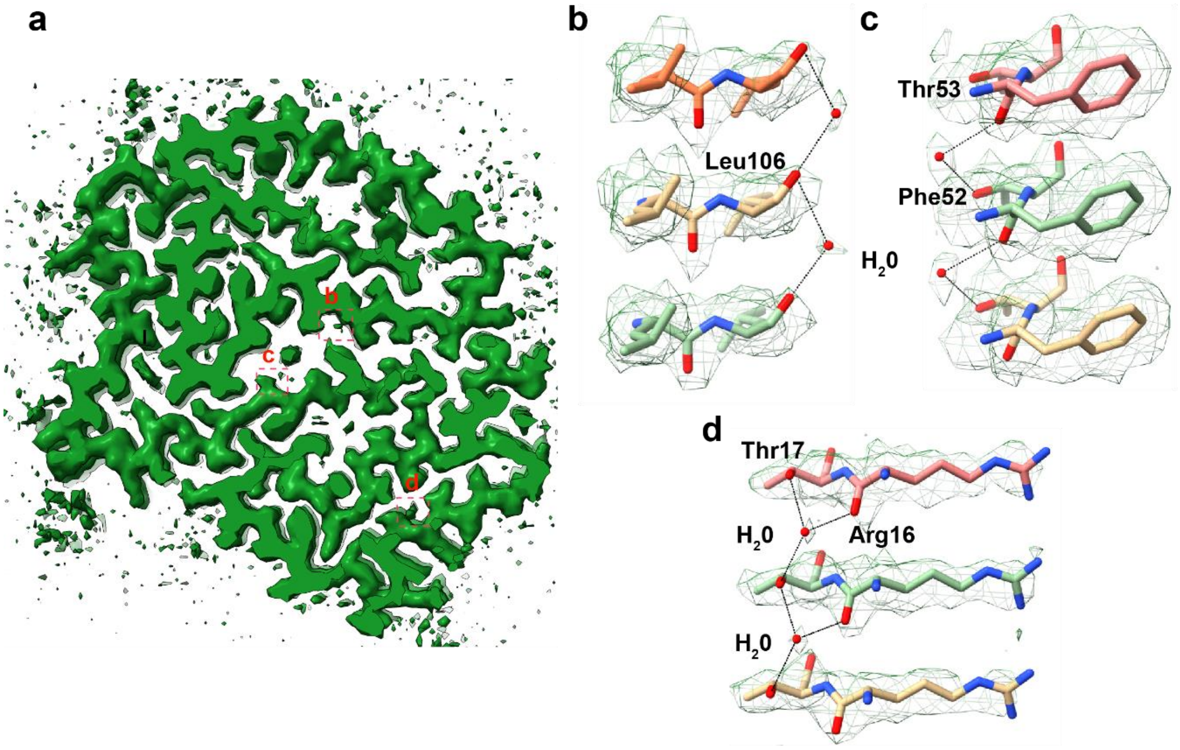

Supplementary Figure 8. **Predicted ordered water molecules within the ALECT2 fibril structure**. **a.** ALECT2 map with predicted water molecules marked in red dotted squares. **b.** Water molecules interacting with Leu106 across the fibril layers. **c.** water molecule interacting with Thr53 and Phe52 across the layers and **d.** water molecule interacting with Thr17 and Arg16 across the fibril layers.

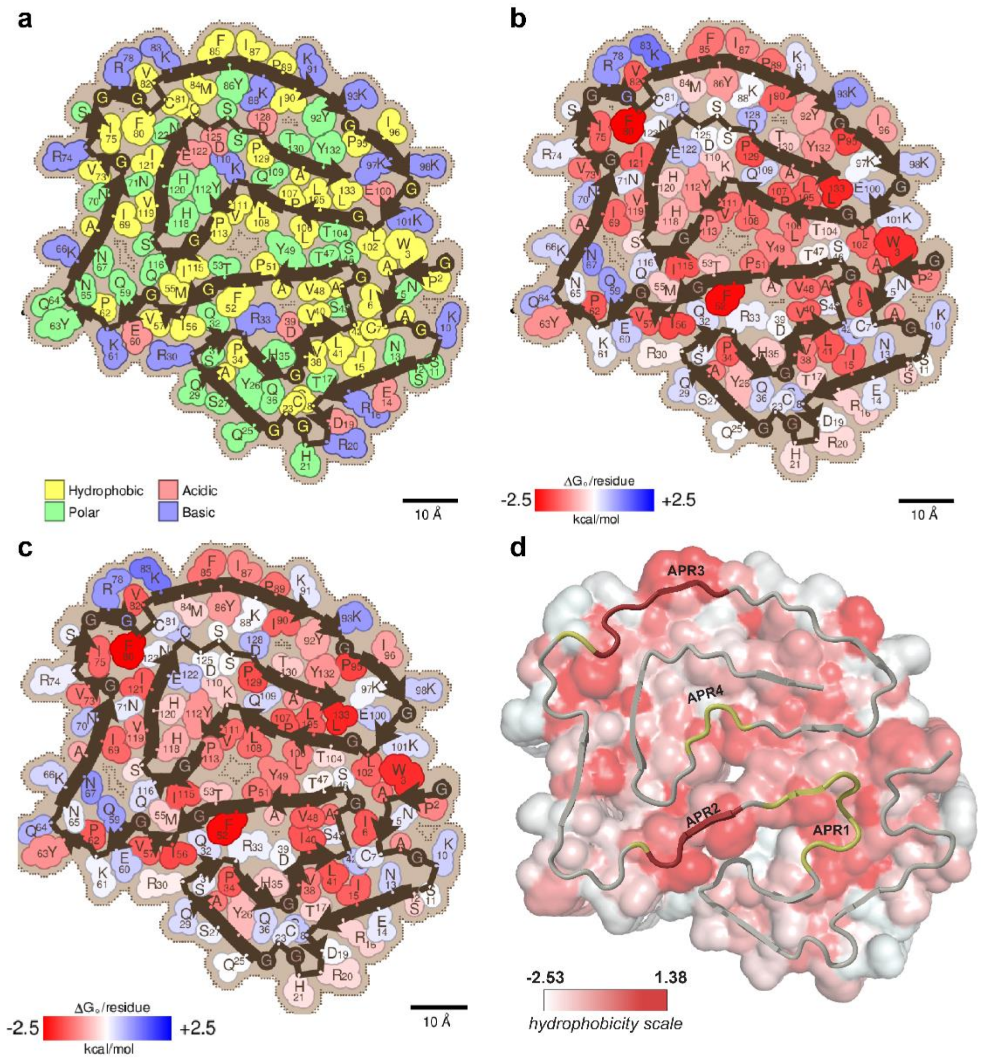

Supplementary Fig. 9. Composition and stability of the ALECT2 fibril core. a. Schematic view of ALECT2 fibrils showing residue composition where residues are color coded by amino acid category, as labeled. **b**. Representation of ALECT2 fibril core depicting stabilizing residues. Strongly stabilizing side chains are colored red, and destabilizing side chains are colored blue. **c.** Solvation energy recalculated after mutating Val40 to Ile, showing minimal difference in local stabilization energy, consistent with the conservative nature of the substitution. **d.** Solvent-accessible surface area (SASA) mapped onto the fibril surface in PyMOL, overlaid with aggregation-prone regions (APRs).

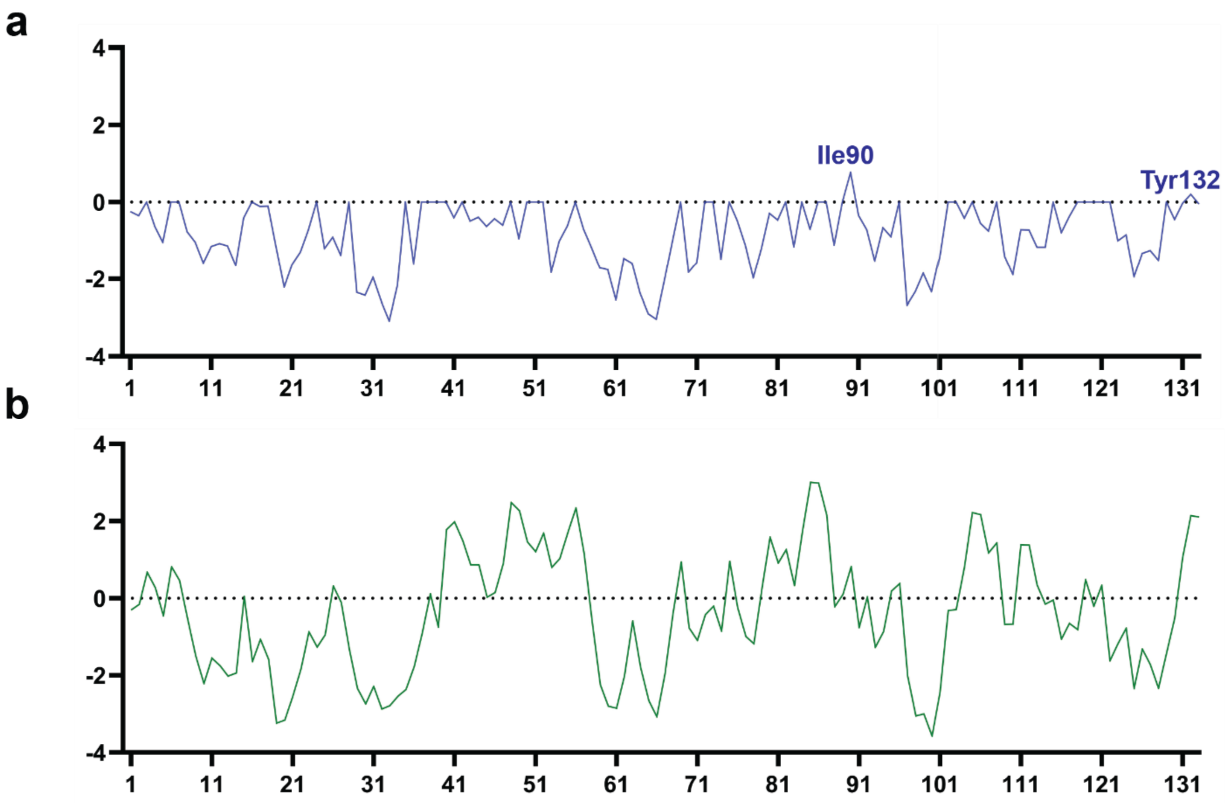

Supplementary Fig. 10. Comparative analysis of aggregation-prone regions (APR) in LECT2 using AggreScan3D 2.0. a. AggreScan3D analysis of the native LECT2 monomer (Chain B, PDB: 5B0H). **b.** Aggrescan3D analysis of the ALECT2 fibril structure (Chain C, PDB: 9NON) from this study. The plots display per-residue aggregation scores, where positive values indicate regions prone to aggregation, and negative values suggest aggregation-resistant regions. Source data provided as part of source data file.

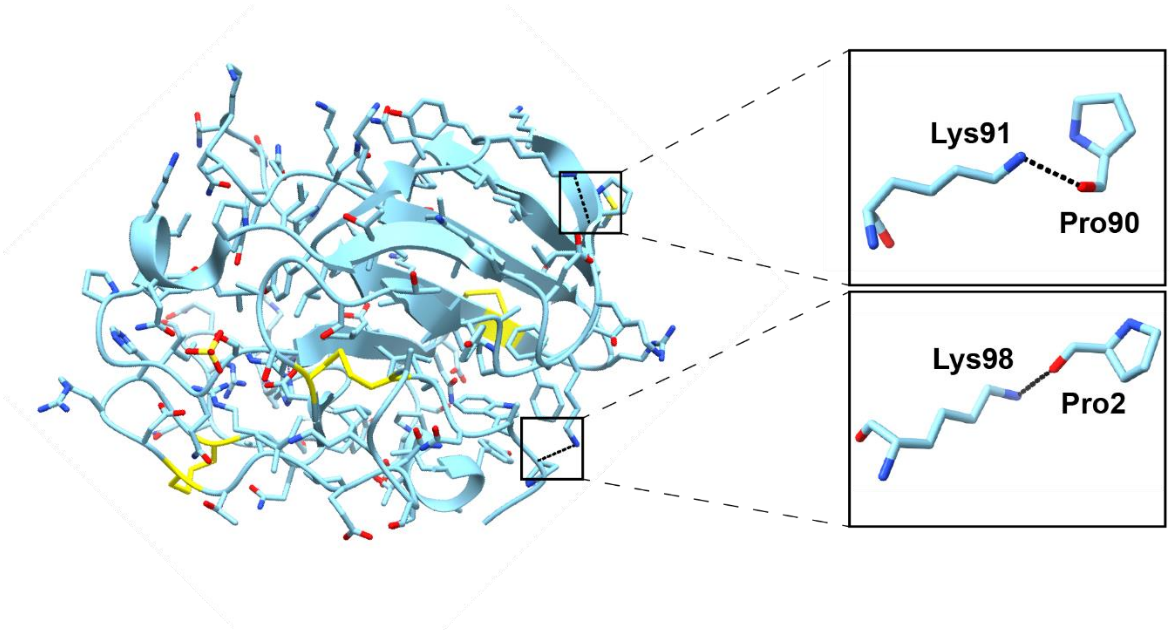

Supplementary Fig. 11. Interaction between lysine residues and the amide backbone of proline in the native LECT2 structure (Chain B, PDB: 5B0H). These interactions help stabilize the native fold and would be disrupted by lysine acetylation, potentially destabilizing the structure and promoting aggregation. Dashed lines, distances lower than 3 Å.

a b

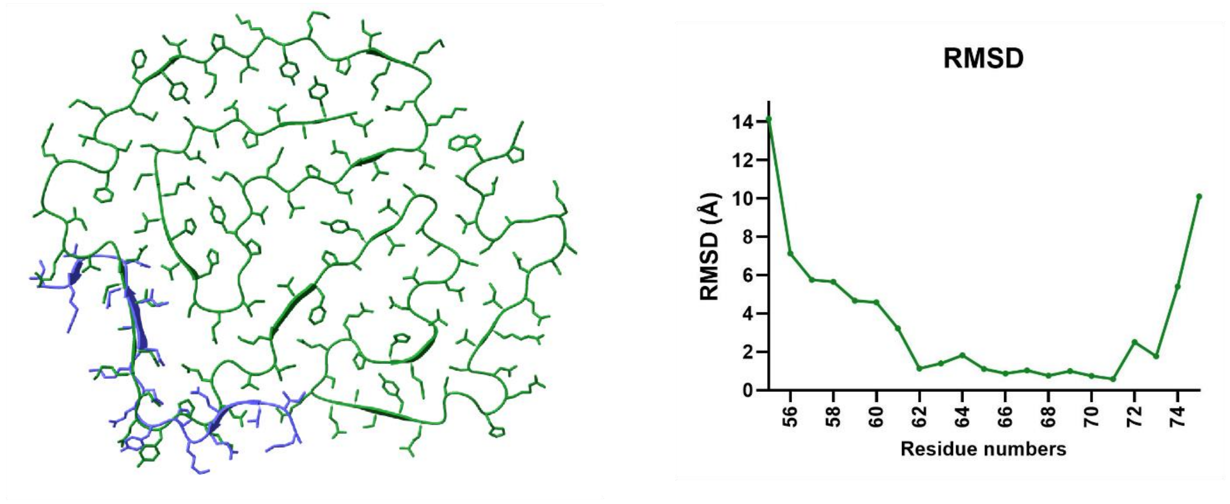

Supplementary Fig. 12. Structural comparison between recombinant LECT2 and *ex-vivo* single protofilament ALECT2 structure from this study. a. *Ex-vivo* single protofilament fibril structure (green, PDBID: 9NON) aligned with the corresponding region of the single protofilament of the recombinant LECT2 structure (blue, PDBID: 8G2V). **b.** RMSD comparison between ALECT2 *ex-vivo* and *in-vitro* fibril structure**.**

Supplementary tables

Peak	Position	Theoretical M_w (Da)	Observed M_w (Da)	PTMs
1	1-133	14566.5	14566.16	3x disulfide bonds
2	1-133	14608.5	14608.23	3x disulfide bonds<br>1x acetylation

Supplementary Table 1. Observed molecular weights from intact protein LC-MS deconvoluted mass spectrum from Supplementary Figure 1. M_w, molecular weight. PTMs, post-translational modifications.

Sequence	Modification	Sequence position in protein	Modified residue
[Y].QNKNAINNGVRISGRGF.[C]	1xAcetyl	[64-80]	K66
[F].YIKPIKY.[K]	1xAcetyl	[86-92]	K91
[Y].KGPIKKGEKL.[G]	1xAcetyl	[93-102]	K98
[F].TGMIVGQEKPY.[Q]	1xAcetyl	[53-63]	K61
[Y].KGPIKKGEKL.[G]	1xAcetyl	[93-102]	K101
[-].GPWANICAGK.[S]*	1xAcetyl [N-term]	[1-10]	G1
[F].TGMIVGQEKPY.[Q]**	1xOxidation	[53-63]	M55
[Y].QNKNAINNGVRISGRGF.[C] **	1x Deamidated	[64-80]	N71
[Y].QNKNAINNGVRISGRGF.[C] **	2x Deamidated	[64-80]	N67, N71

***Indicates a low-abundance N-terminally acetylated peptide.**

**** We detected low-abundance hits for Asn/Gln deamidation, but we could not confidently confirm these by the intact mass analysis due to the small mass shifts (1 Da) and overlap with naturally occurring isotope peaks. A single methionine oxidation (Met 55) was observed as well, though both methionine oxidation and deamidation can occur during sample preparation, for example, prolonged incubations at neutral pH or oxidative handling conditions are known to cause these changes[1,2]. Thus, while we cannot exclude their presence *in vivo*, we interpret these modifications cautiously and regard them as potential preparation artifacts rather than as defining features of the fibrils.**

Supplementary Table 2. LC-MS/MS analysis of chymotrypsin-digested ALECT2 fibrils extracted from the kidney reveals multiple lysine acetylation sites.

Supplementary Table 3: Cryo-EM data collection, refinement and validation statistics

	Single (EMD-49601) (PDB 9non)	Double-C1 (EMDB-49624)	Double-C2 (EMDB-49623)
**Data collection and processing**			
Magnification		130,000x	
Voltage (kV)		300	
Electron exposure (e–/Å²)		40	
Exposure Time (sec)		3.69	
Number of frames		40	
Defocus range (μm)		-0.8 to -2.6	
Pixel size (Å)		0.954	
Symmetry imposed		C1	
Micrographs collected		22,899	
Micrographs processed	14,112	11,943	11,943
Total particles	1,840,366 (box 300)	197,045 (box 360)	197,045 (box 360)
Particles used in final map	36,221	44,668	9,848
Helical parameters			
Twist	-0.91	-0.80	-0.81
Rise	4.75	4.75	4.75
Map resolution (Å)	2.4	5.0	5.6
FSC threshold	0.143	0.143	0.143
Map resolution range (Å)	2.26 – 2.85	n/a	n/a
**Refinement**			
Model resolution (Å)	2.1		
FSC threshold	0.143		
Model resolution range (Å)	2.1 – 2.4		
Map sharpening *B* factor (Å²)	64.76		
Model composition			
Chains	5		
Non-hydrogen atoms	0		
Protein residues	665		
Ligands	0		
B factors (Å²)			
Protein	43.29		
Ligand	0		
R.m.s. deviations			
Bond lengths (Å)	0.004		
Bond angles (°)	1.004		
Validation			
MolProbity score	1.61		
Clashscore	2.95		
Poor rotamers (%)	0.90		
Ramachandran plot			
Favored (%)	90.84		
Allowed (%)	9.16		
Disallowed (%)	0.00		

Supplementary References

1 Bettinger, J. Q., Welle, K. A., Hryhorenko, J. R. & Ghaemmaghami, S. Quantitative Analysis of in Vivo Methionine Oxidation of the Human Proteome. *J Proteome Res* **19**, 624-633 (2020). https://doi.org/10.1021/acs.jproteome.9b00505

2 Liu, S., Moulton, K. R., Auclair, J. R. & Zhou, Z. S. Mildly acidic conditions eliminate deamidation artifact during proteolysis: digestion with endoprotease Glu-C at pH 4.5. *Amino Acids* **48**, 1059-1067 (2016). https://doi.org/10.1007/s00726-015-2166-z
