# Supplementary material for: Structural polymorphism of ex-vivo ALECT2 amyloid fibrils revealed by cryo-EM
Source: Nat Commun. 2026 Apr 11;17:5108. doi: 10.1038/s41467-026-71223-3 (PMC13247027; doi:10.1038/s41467-026-71223-3)
Supplement: Supplementary file 3 — Transparent Peer Review file [file 41467_2026_71223_MOESM3_ESM.pdf]

nature portfolio

Peer Review File

Structural polymorphism of ex-vivo ALECT2 amyloid fibrils revealed by cryo-EM

Corresponding Author: Dr Lorena Saelices Gomez

This file contains all reviewer reports in order by version, followed by all author rebuttals in order by version.

Version 0:

Reviewer comments:

Reviewer #1

(Remarks to the Author)
The article by Afrin et al. reports the first structure of fibrils derived from ALECT2, a systemic amyloidosis affecting the kidney. The three dimensional structure of the ALECT2 fibril core is revealed with sufficient clarity to indicate backbone geometry and the presence of buried disulfide bridges. It also confirms a homozygous sequence polymorphism at position 40. While the density does not directly reveal the positions of acetylated lysine residues, these are mapped by mass spectrometry. Stabilizing molecular interactions are described within the fibril core, and appear to match those observed in other amyloid fibrils including polar ladders across staggered fibril layers.

Given the novelty of this structure and its implications for the field of systemic amyloidosis, and ALECT2 in particular, I am enthusiastic about its publication, but urge the authors to consider the questions and points raised below.

Major questions:

Since ALECT2 is a relatively recently characterized amyloidosis, but has a known disease-correlated sequence polymorphism, I40V, and since the structure determined was from a patient homozygous for Valine at position 40, do the authors expect the structure determined to be the same for heterozygous patients or those homozygous for Isoleucine at that position?

It is intriguing that the patient harboring the fibrils whose structure was determined also had a co-occurring carcinoma. Are the authors able to discriminate the effects of the carcinoma on the structure of the fibrils? In other words, are these fibrils expected of ALECT2 patients that did not suffer renal carcinoma, or are they a unique presentation of this condition?

How does MS indicate the acetylation is integrated into fibrils, not just in free monomers or oligomers (e.g. the multimeric species identified by western blot)? On this point, it would be helpful to verify that the chymotrypsin digestion used to detect the acetylation was indeed successful on the fibrils.

Although the double protofilament type 1 appears to be 2 fold symmetric, the type 2 protofilament structure seems asymmetric. In each case, the correlation of the model to the map for each protofilament would be a useful metric to know. It might also help indicate whether all protofilament structures described by the lower resolution maps match the single protofilament fold equally well.

Minor questions:

Although the putative fibril cofactors could not be identified on the basis of their densities, it would help if those densities were labeled or in some way catalogued and if their respective volumes were also estimated.

Rather than labeling certain waters as stabilizing, it would be more clear to label them as 'ordered waters'.

It would help if the fibril core structure shown in Fig 2C had more residues numbered, including the location of the I40V polymorphism.

The double protofilaments in Figure 4 are described as being related to each other by inversion, but the operation appears to be a simple rotation (of approximately 180 degrees) about the fibril growth axis.

It would be helpful to include in Sup. Table 3 the refined helical parameters of the structures and for the text to note how different these were across the different protofilament types.

The energetics maps displayed (Figure S8) do not clearly indicate what regions of the fibril core are more stabilizing than others. Would it be possible for the authors to combine the information from their aggregation propensity predictions and the energetics estimates to better indicate how these two correlate?

In Figure 5b, it would be helpful to have the beta strands in the schematic numbered or labeled for both the native and fibril folds.

Reviewer #2

(Remarks to the Author)
This manuscript reports the high-resolution Cryo-EM structure of ex vivo kidney ALECT2 fibrils from an affected patient. Besides defining the structure of the most prevalent single protofilament, the results show the presence of less abundant double protofilament morphologies. This is the first resolved natural ALECT fibril structure and provides important novel molecular knowledge, fundamental to cast light on the unfolding process and dynamics in tissues. Even more so, considering that the structure of in vitro ALECT2 fibrils does not fully match the natural one.

In addition to providing the fibril structure, the authors perform an MS-based characterization of the amyloid extract, to confirm LECT2 sequence and to explore the presence of PTMs. In the undigested extract, they observe a major deconvoluted peak in line with full-length I40V ALECT2 with intact disulphide bridges, and several additional peaks of higher molecular mass, consistent with PTMs-containing LECT2 proteoforms. The most intense such peak is consistent with monoacetylated LECT2. Presence of acetylation on several K residues, surface-exposed on the fibril structure, has then been confirmed by high-resolution MS/MS analysis. It is not clear to me, instead, if acetylation of the N terminus, frequent in eukaryotic proteins and potentially accounting for a significant fraction of LECT2 proteoforms forming the +42 Da peak, has been explored (it is not indicated in the methods). Is the presence of K acetylation compatible with the densities observed by Cryo-EM?

Given the emphasis on PTMs in the manuscript, it must be noted that other potentially relevant PTMs have not been considered. The authors should comment on the additional peaks in the deconvoluted spectrum. Amyloid fibrils from various proteins have been reported to contain multiple potentially destabilizing PTMs, e.g. extensive oxidation and deamidation. Have they been searched for?

Overall, although opening an interesting scenario, the considerations on the potential role of acetylation in LECT2 amyloidogenesis are currently speculative. The timing of acetylation is in fact unclear, no information is available on this PTM in soluble ALECT2 in diseased and healthy individuals, the actual distribution of the acetyl groups on the various K residues or on the N-terminus is undefined (addition of a single acetyl group per LECT2 molecule seems the most frequent case), and no clear experimental mapping of this PTMs on the structure is presented.

Version 1:

Reviewer comments:

Reviewer #1

(Remarks to the Author)
The authors have significantly improved their manuscript by systematically addressing each of the reviewer comments. Overall, the author responses satisfactorily addressed most of my questions and suggestions. However, I would like to note that despite the middling resolutions observed for their double protofilament structures, cited at 5 and 5.6Å, the authors could still more robustly characterize the map to model fit. Rather than appearing misleading, the metrics used, e.g. fit scores from molecular dynamics or Rosetta modeling to cryoem maps (https://pubs.acs.org/doi/10.1021/ct500995d) and Q-score analyses (https://www.nature.com/articles/s41592-020-0731-1) would be informative of overall map and model quality. Some of these tools also permit fitting to variably resolved areas of a map.

Reviewer #2

(Remarks to the Author)
The authors have addressed the concerns raised during the first revision.

Minor observations:
- given that they mention a 200-fold lower intensity of the acetylated compared to the non-acetylated N-terminal peptide, I suggest specifying what this calculation is based on (PSM, XIC etc).

- In my opinion, the following considerations could be moved to the supplementary material: “We also detected low-abundance hits for Asn/Gln deamidation, but we could not confidently confirm these by the intact mass analysis due to the small mass shifts (1 Da) and overlap with naturally occurring isotope peaks. A single methionine oxidation (Met 55) was observed as well, though both methionine oxidation and deamidation can occur during sample preparation, for example, prolonged incubations at neutral pH or oxidative handling conditions are known to cause these changes. Thus, while we cannot exclude their presence in vivo, we interpret these modifications cautiously and regard them as potential preparation artifacts rather than as defining features of the fibrils”

Version 2:

Reviewer comments:

Reviewer #1

(Remarks to the Author)
All my concerns have been addressed.

Open Access This Peer Review File is licensed under a Creative Commons Attribution 4.0 International License, which permits use, sharing, adaptation, distribution and reproduction in any medium or format, as long as you give appropriate credit to the original author(s) and the source, provide a link to the Creative Commons license, and indicate if changes were made.
In cases where reviewers are anonymous, credit should be given to 'Anonymous Referee' and the source.
The images or other third party material in this Peer Review File are included in the article’s Creative Commons license, unless indicated otherwise in a credit line to the material. If material is not included in the article’s Creative Commons license and your intended use is not permitted by statutory regulation or exceeds the permitted use, you will need to obtain permission directly from the copyright holder.
To view a copy of this license, visit https://creativecommons.org/licenses/by/4.0/

REVIEWER COMMENTS

Reviewer #1 (Remarks to the Author):

The article by Afrin et al. reports the first structure of fibrils derived from ALECT2, a systemic amyloidosis affecting the kidney. The three dimensional structure of the ALECT2 fibril core is revealed with sufficient clarity to indicate backbone geometry and the presence of buried disulfide bridges. It also confirms a homozygous sequence polymorphism at position 40. While the density does not directly reveal the positions of acetylated lysine residues, these are mapped by mass spectrometry. Stabilizing molecular interactions are described within the fibril core, and appear to match those observed in other amyloid fibrils including polar ladders across staggered fibril layers.

Given the novelty of this structure and its implications for the field of systemic amyloidosis, and ALECT2 in particular, I am enthusiastic about its publication, but urge the authors to consider the questions and points raised below.

Major questions:
1. Since ALECT2 is a relatively recently characterized amyloidosis, but has a known disease-correlated sequence polymorphism, I40V, and since the structure determined was from a patient homozygous for Valine at position 40, do the authors expect the structure determined to be the same for heterozygous patients or those homozygous for Isoleucine at that position?

Response: We thank the reviewer for raising this important point. We are not aware of any confirmed cases of patients homozygous for isoleucine at position 40 (I40/I40) in ALECT2. Furthermore, existing clinical data indicate that the V40 allele is strongly associated with disease risk and is required for pathogenesis[1]. We note, however, that there is at least one documented heterozygous I40/V40 case. In that context, we speculate that the fibril fold would be similar because the Ile40 to Val40 mutation is conservative with respect to size and hydrophobicity, as well as compatible with the fibril fold.
Structurally, the I40V substitution slightly weakens local hydrophobic packing (Reviewer Figure 1a), leading to a marginally lower estimated solvation energy for the Val40 form (–67.9 kcal/mol per layer) compared to the Ile40 form (–68.2 kcal/mol per layer), assuming similar backbone conformation (Reviewer figure 1b-c). This difference is minimal and unlikely to affect the overall fibril structure or stability.

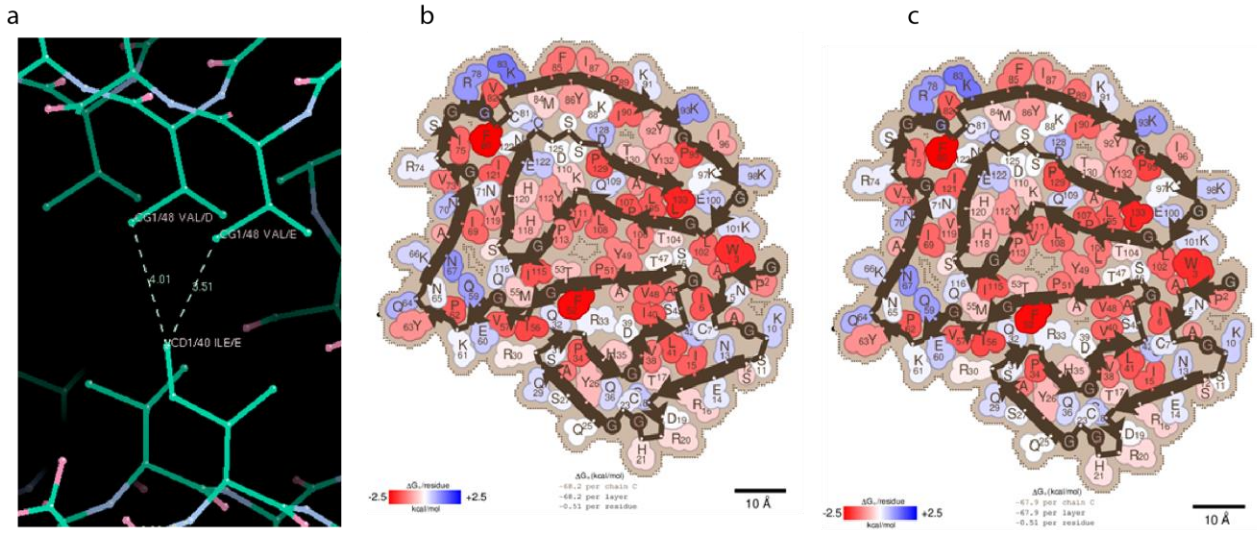

Reviewer figure 1. (a) depiction of hydrophobic interactions between Ile40 (layer n) and Val48 of layer n and n+1. Free energy of solvation calculated for (b) Ile40 form and (c) Val40 form. Strongly stabilizing side chains are colored red, and destabilizing side chains are colored blue.

Similarly, APR prediction using Aggrescan3D showed nearly identical profiles with only a minimal increase in the score for Ile40 form (Reviewer Figure 2). Overall, these findings suggest that the I40V mutation has little impact

on the final fibril structure and its stability. It is possible that the mutation has more influence on the native form of LECT2. The I40V mutation is believed to increase the conformational heterogeneity of the zinc free form of LECT2[1].

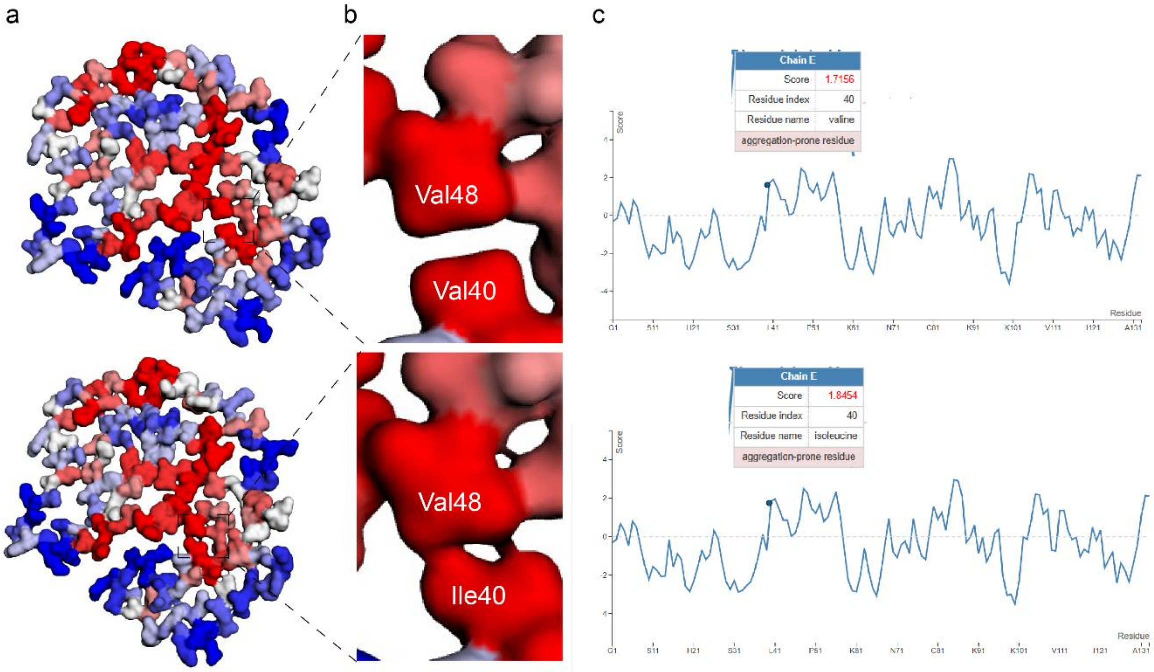

Reviewer Figure 2. (a) Aggrescan3D visual representation of the aggregation score for Val40 (top) and Ile40 (bottom). The residues are colored according to their score, ranging from blue, which implies a soluble residue, to various red shades indicating aggregation prone residues. (b) zoomed in view of the original Val40 form (upper panel) and Ile40 form (created by mutating the structure of the Val40 form (lower panel). (c) Aggrescan3D analysis plot and score of the original Val40 (upper) form and mutated Ile40 (lower) form. The plots display per-residue aggregation scores, where positive values indicate regions prone to aggregation, and negative values suggest aggregation-resistant regions.

In light of this comment, we have now updated the manuscript discussion, which now reads "Indeed, the analysis of the solvation energy of the *ex-vivo* ALECT2 amyloid structure when incorporating an isoleucine in position 40 shows little to no change in the estimated structure stability (Supplementary Fig. 8c). Thus, the *ex-vivo* ALECT2 amyloid fold could be compatible with both residues." We have included a panel in the Supplementary Fig. 8c with the free energy of solvation of the *ex-vivo* ALECT2 amyloid structure with an isoleucine in the residue position 40.

2. It is intriguing that the patient harboring the fibrils whose structure was determined also had a co-occurring carcinoma. Are the authors able to discriminate the effects of the carcinoma on the structure of the fibrils? In other words, are these fibrils expected of ALECT2 patients that did not suffer renal carcinoma, or are they a unique presentation of this condition?

Response: We thank the reviewer for this thoughtful question. In this patient, amyloid deposition was clinically evident at age 54, whereas the renal carcinoma was not identified until age 61, at the time of nephrectomy[2]. This timeline strongly suggests that amyloidogenesis preceded the carcinoma, making it unlikely that the tumor influenced the fibril fold we report.

Studies have reported that about 12.5% of ALECT2 patients are also diagnosed with carcinoma, most often discovered incidentally at the time of nephrectomy. In these cases (including in our patient), the amyloid deposits are consistently located in the non-neoplastic portion of the kidney, rather than within the tumor itself[2,3]. This separation further supports that the fibril structure is unlikely to be influenced by the carcinoma. We therefore expect that the conformation observed here is representative of ALECT2 patients without carcinoma, although additional analyses of fibrils from patients with and without comorbidities will be important to confirm this

conclusion. We have noted this in the revised Discussion, which now reads "For instance, in the patient analyzed here, the co-occurrence of carcinoma might have influenced fibril formation; however, because the cancer developed after amyloid deposition, this seems unlikely."

3. How does MS indicate the acetylation is integrated into fibrils, not just in free monomers or oligomers (e.g. the multimeric species identified by western blot)? On this point, it would be helpful to verify that the chymotrypsin digestion used to detect the acetylation was indeed successful on the fibrils.

Response: We thank the reviewer for this observation. We have now included the following to the Materials and Methods section: "Our purification protocol is biased towards amyloid fibrils; therefore, we do not expect significant monomers or oligomers to be present in the fibril preparation. Although we cannot completely rule out the presence of a small amount of monomer or oligomer, the influence of these would likely be considered spectra noise. The chymotrypsin digestion performed on the sample showed chymotryptic peptides mapped to the entire LECT2 protein, confirming the success of the digestion."

4. Although the double protofilament type 1 appears to be 2-fold symmetric, the type 2 protofilament structure seems asymmetric. In each case, the correlation of the model to the map for each protofilament would be a useful metric to know. It might also help indicate whether all protofilament structures described by the lower resolution maps match the single protofilament fold equally well.

Response: We thank the reviewer for this helpful suggestion and have included the following information into the Results section. "The final resolutions of the double protofilament type 1 and type 2 maps are 5.0 Å and 5.6 Å, respectively. At such resolutions, fibril backbone and side chain features are not well resolved, preventing reliable model building and accurate calculation of the model to the map correlation. For this reason, we did not perform de novo modeling, but instead rigid body docked the high resolution protofilament structure into these lower resolution densities (Fig. 4a). Given the limited resolution and the presence of unresolved densities in both the type 1 and type 2 double protofilament maps, any quantitative metrics would be unreliable and potentially misleading. Visual inspection of the fit indicates that the backbone of single protofilament model docks reasonably well within the protofilament density of the double protofilament maps although local variations very likely exist (Fig. 4a)."

Minor questions:

1. Although the putative fibril cofactors could not be identified on the basis of their densities, it would help if those densities were labeled or in some way catalogued and if their respective volumes were also estimated.

Response: We do observe extra densities in the reconstructed maps at lower contour levels, suggesting the presence of non-proteinaceous cofactors. However, at the higher contour levels used for reliable model building, these densities become poorly defined due to low signal-to-noise and appear hollow. In the post-processed maps, the signal quality was further reduced, which prevents us from confidently interpreting these features. Additionally, while we attempted to estimate the volumes of the extra densities, this proved challenging because some of these features appear continuous across multiple fibril layers, whereas others are localized to individual layers. Without precise information on the number of repeating layers in the fibril, it is not possible to accurately define the volume of these densities. For this reason, we believe adding this information to the paper would be too speculative. However, to address our curiosity and that of this reviewer, we calculated the projected area of each density at the cross-sectional level, which provides a two-dimensional measure within the available data, which are included here for the reviewer's consideration.

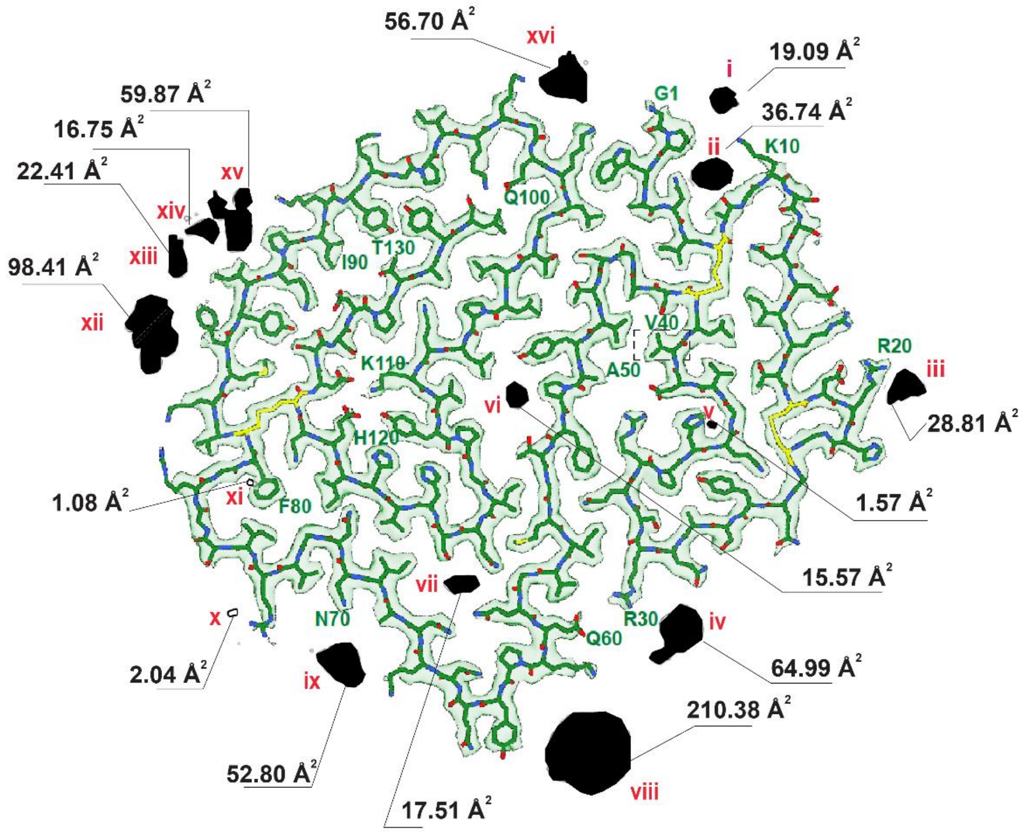

Reviewer Figure 3. Quantification of extra densities observed in the ALECT2 single-protofilament structure. Cryo-EM density map of the ALECT2 single protofilament overlaid with the atomic model, showing non-proteinaceous extra densities (black). Each density was assigned a Roman numeral label (i–xvi), and its projected cross-sectional area was estimated in Å^2.

2. Rather than labeling certain waters as stabilizing, it would be more clear to label them as 'ordered waters'.

Response: Thank you for this suggestion. These waters are now referred to as ordered waters in the results and figure legend for Supplementary Fig 7.

3. It would help if the fibril core structure shown in Fig 2C had more residues numbered, including the location of the I40V polymorphism.

Response: Thank you for suggesting this. We have now numbered every tenth residues and also labeled the mutated residue.

4. The double protofilaments in Figure 4 are described as being related to each other by inversion, but the operation appears to be a simple rotation (of approximately 180 degrees) about the fibril growth axis.

Response: We thank the reviewer for this correction. We have now changed the description from inversion to rotation where applicable.

5. It would be helpful to include in Sup. Table 3 the refined helical parameters of the structures and for the text to note how different these were across the different protofilament types.

Response: We missed this information in the stats table, thank you for your observation. It is now included in the stats table as well as the results.

6. The energetics maps displayed (Figure S8) do not clearly indicate what regions of the fibril core are more

stabilizing than others. Would it be possible for the authors to combine the information from their aggregation propensity predictions and the energetics estimates to better indicate how these two correlate?

Response: We thank the reviewer for this insightful comment. We have added an updated panel to Supplementary Figure 8 (d) highlighting the overlap between APRs and regions of low solvation energy, and we describe this correlation in both the results and discussions as follows.

The Results section now reads "To further evaluate the contribution of these regions to fibril stability, we analyzed solvent-accessible surface area and solvation energy across the fibril (Supplementary Fig. 8d), which revealed that APR1, APR2, and APR4 form a hydrophobic and energetically stable core consistent with our aggregation-propensity predictions."

The Discussion section now reads "Our solvent-accessible surface area and solvation energy analyses (Supplementary Fig. 8d) showed that regions corresponding to APR1, APR2, and APR4 formed a hydrophobic and energetically stable core, exhibiting lower solvation energy and reduced surface exposure relative to other fibril regions. These observations were consistent with our aggregation-propensity predictions (Fig. 5a). Together, these findings suggested that the APRs corresponded to regions contributing most significantly to fibril stabilization."

7. In Figure 5b, it would be helpful to have the beta strands in the schematic numbered or labeled for both the native and fibril folds.

Response: We have now included the β strand numbering for both the native and fibril fold as suggested.

Reviewer #2 (Remarks to the Author):

This manuscript reports the high-resolution Cryo-EM structure of ex vivo kidney ALECT2 fibrils from an affected patient. Besides defining the structure of the most prevalent single protofilament, the results show the presence of less abundant double protofilament morphologies. This is the first resolved natural ALECT fibril structure and provides important novel molecular knowledge, fundamental to cast light on the unfolding process and dynamics in tissues. Even more so, considering that the structure of in vitro ALECT2 fibrils does not fully match the natural one.

In addition to providing the fibril structure, the authors perform an MS-based characterization of the amyloid extract, to confirm LECT2 sequence and to explore the presence of PTMs.

1. In the undigested extract, they observe a major deconvoluted peak in line with full-length I40V ALECT2 with intact disulphide bridges, and several additional peaks of higher molecular mass, consistent with PTMs-containing LECT2 proteoforms. The most intense such peak is consistent with monoacetylated LECT2. Presence of acetylation on several K residues, surface-exposed on the fibril structure, has then been confirmed by high-resolution MS/MS analysis. It is not clear to me, instead, if acetylation of the N terminus, frequent in eukaryotic proteins and potentially accounting for a significant fraction of LECT2 proteoforms forming the +42 Da peak, has been explored (it is not indicated in the methods). Is the presence of K acetylation compatible with the densities observed by Cryo-EM?

Response: Based on the reviewer's suggestion we included protein N-terminal acetylation as part of the search protocol. In the tryptic dataset, an N-terminally acetylated peptide was identified but at more than 200-fold lower abundance than the non-acetylated version, suggesting that if present, N-terminal acetylation represents only a very minor fraction of the molecules.

We added the following clarification to the Results: "A low-abundance N-terminally acetylated peptide was detected though its intensity was more than 200-fold lower than that of the corresponding non-acetylated peptide, indicating that N-terminal acetylation, if present, occurred only in a very small fraction of molecules (Supplementary Table 2)." We also added this PTM to Supplementary Table 2.

2. Is the presence of K acetylation compatible with the densities observed by Cryo-EM?

Response: Densities are not compatible with lysine acetylation.

3. Given the emphasis on PTMs in the manuscript, it must be noted that other potentially relevant PTMs have not been considered. The authors should comment on the additional peaks in the deconvoluted spectrum. Amyloid fibrils from various proteins have been reported to contain multiple potentially destabilizing PTMs, e.g. extensive oxidation and deamidation. Have they been searched for?

Response: We originally searched for phosphorylation, acetylation, methylation, dimethylation, trimethylation, and ubiquitination, and consistently identified acetylation. Based on reviewer feedback, we extended our searches to include methionine oxidation and Asn/Gln deamidation. Now the results section includes the following "We also detected low-abundance hits for Asn/Gln deamidation, but we could not confidently confirm these by the intact mass analysis due to the small mass shifts (1 Da) and overlap with naturally occurring isotope peaks. A single methionine oxidation (Met 55) was observed as well, though both methionine oxidation and deamidation can occur during sample preparation, for example, prolonged incubations at neutral pH or oxidative handling conditions are known to cause these changes14,15. Thus, while we cannot exclude their presence in vivo, we interpret these modifications cautiously and regard them as potential preparation artifacts rather than as defining features of the fibrils." We also added these PTMs to Supplementary Table 2.

4. Overall, although opening an interesting scenario, the considerations on the potential role of acetylation in LECT2 amyloidogenesis are currently speculative. The timing of acetylation is in fact unclear, no information is available on this PTM in soluble ALECT2 in diseased and healthy individuals, the actual distribution of the acetyl groups on the various K residues or on the N-terminus is undefined (addition of a single acetyl group per LECT2 molecule seems the most frequent case), and no clear experimental mapping of this PTMs on the structure is presented.

Response: We thank the reviewer for their input. We agree that the current evidence is preliminary. We acknowledge that our observations on lysine acetylation in ALECT2 fibrils are exploratory and do not yet define the timing, frequency, or distribution of this PTM.

However, what we do know from our data is the following:

- Intact mass spectrometry revealed that the predominant modified species carried a single lysine acetylation, while chymotrypsin-based MS/MS analysis identified multiple potential acetylation sites, indicating that different lysines can be acetylated across the fibril population, though typically only one per monomer (Supplementary Table 2).
- Importantly, because these data were obtained from fibril extracts, where soluble monomeric LECT2 does not copurify, our analyses reflect modifications present in fibril-associated LECT2 rather than soluble LECT2 species.

Based on this comment we clarified our language in the Discussion, which now reads "Given the limited experimental evidence and the preliminary nature of our findings, it remains challenging to determine the timing, frequency and distribution of acetylation or its specific role in ALECT2 amyloidosis. Further exploration is necessary to understand how this modification influences protein-protein interactions and whether it plays a functional role in the pathogenesis of ALECT2 amyloidosis."

References:

1 Ha, J. H., Tu, H. C., Wilkens, S. & Loh, S. N. Loss of bound zinc facilitates amyloid fibril formation of leukocyte-cell-derived chemotaxin 2 (LECT2). *J Biol Chem* **296**, 100446 (2021). https://doi.org/10.1016/j.jbc.2021.100446

2 Benson, M. D., James, S., Scott, K., Liepnieks, J. J. & Kluve-Beckerman, B. Leukocyte chemotactic factor 2: A novel renal amyloid protein. *Kidney Int* **74**, 218-222 (2008). https://doi.org/10.1038/ki.2008.152

3 Said, S. M. *et al.* Characterization and outcomes of renal leukocyte chemotactic factor 2-associated amyloidosis. *Kidney Int* **86**, 370-377 (2014). https://doi.org/10.1038/ki.2013.558

REVIEWER COMMENTS

Reviewer #1 (Remarks to the Author):

The authors have significantly improved their manuscript by systematically addressing each of the reviewer comments. Overall, the author responses satisfactorily addressed most of my questions and suggestions. However, I would like to note that despite the middling resolutions observed for their double protofilament structures, cited at 5 and 5.6Å, the authors could still more robustly characterize the map to model fit

Response: We appreciate the reviewer's suggestion. The double protofilament maps are of low resolution and display limited structural detail including low signal-to-noise, hollow interior features, lack of side-chain information, and non-separated β-strand layers (see Extra Fig. 1). To explore whether any meaningful model fitting, we first clipped the maps into thin Z-sections using ChimeraX, and then using COOT to perform manual docking of the Cα backbone into the densities followed local Rigid Body Fit Zone. We also attempted automated model optimization using Phenix. For the double protofilament 2 map (5.6 Å), all refinement attempts failed to improve the fit and consistently produced models worse than the original manually fitted version (Extra Fig. 2a). The estimated model-to-map Q-score for double protofilament 2 was 0.16, consistent with the map's severe limitations. In contrast, refinement of the double protofilament 1 map (5 Å) yielded a Q-score of 0.42, reflecting interpretable correlation of model to map (Extra Fig. 2b).

Based on these observations, the Results section now reads as follows:

"The final resolutions of the double protofilament 1 and 2 maps are 5.0 Å and 5.6 Å, respectively. At such resolutions, the backbone density appears hollow with undefined regions and the side chain features are not well resolved, preventing reliable model building and accurate calculation of the model-to-map correlation. Thus, we did not perform *de novo* modeling, but instead rigid body docked the high-resolution single protofilament structure into these lower resolution densities (Fig. 4a). We obtained a Q-score of 0.42 from the map of the double protofilament 1, reflecting interpretable correlation of model-to-map. In contrast, the map of the double protofilament 2 produced only a Q-score of 0.16, consistent with its lower resolution at 5.6 Å. Visual inspection of the fit indicates that the backbone of the single protofilament model docks reasonably well within the protofilament density of the double protofilament maps although local variations very likely exist (Fig. 4a)."

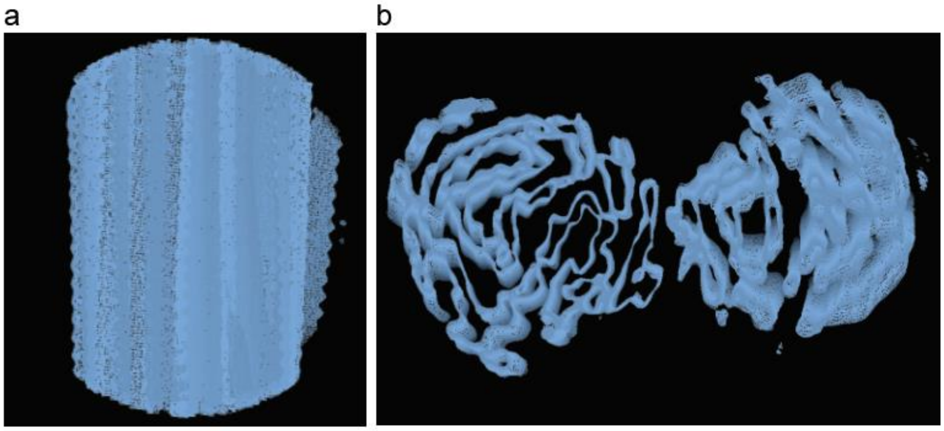

Extra figure 1: Side view (a) and top view (b) of the double protofilament 2.

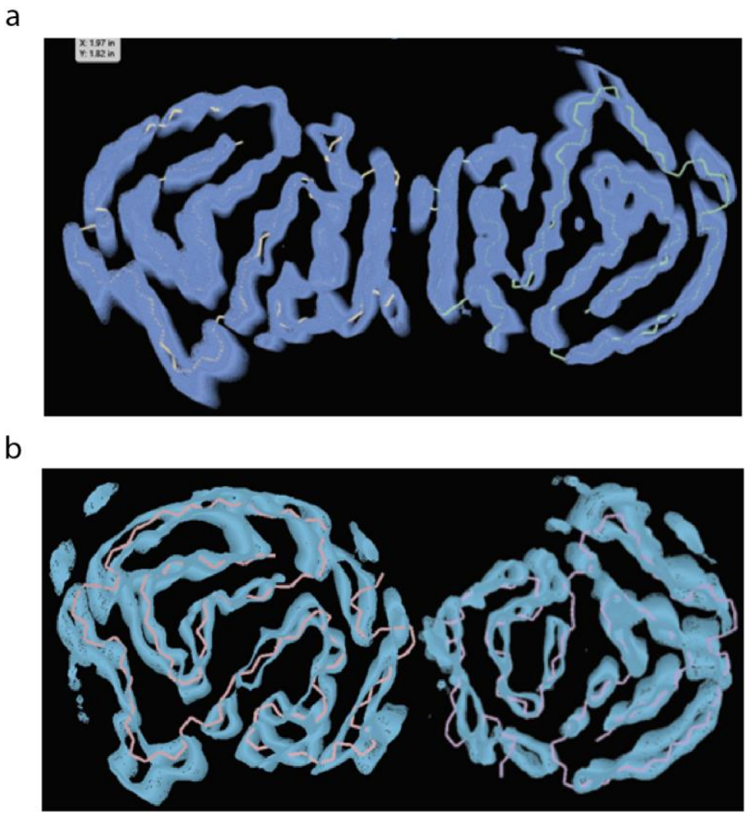

Extra figure 2: Rigid body fitting of a single-layer model of the single protofilament into the density map of double protofilament 1 (a) and 2 (b).

Reviewer 2

Minor observations

1- given that they mention a 200-fold lower intensity of the acetylated compared to the non-acetylated N-terminal peptide, I suggest specifying what this calculation is based on (PSM, XIC etc).

Response: We thank the reviewer for this suggestion. We have now specified the what the calculation is based on and it now reads "A low-abundance N-terminally acetylated peptide was detected, but its signal intensity was >200-fold lower than that of the corresponding non-acetylated peptide based on extracted ion chromatograms

(XIC). This indicates that N-terminal acetylation, if present, occurs only in a very small fraction of molecules (Supplementary Table 2)."

2- In my opinion, the following considerations could be moved to the supplementary material: "We also detected low-abundance hits for Asn/Gln deamidation, but we could not confidently confirm these by the intact mass analysis due to the small mass shifts (1 Da) and overlap with naturally occurring isotope peaks. A single methionine oxidation (Met 55) was observed as well, though both methionine oxidation and deamidation can occur during sample preparation, for example, prolonged incubations at neutral pH or oxidative handling conditions are known to cause these changes. Thus, while we cannot exclude their presence in vivo, we interpret these modifications cautiously and regard them as potential preparation artifacts rather than as defining features of the fibrils"

Response: We thank the reviewer for their suggestion. We have now moved this information to the supplementary table 2.
